# Cornea modelling

**DOI:** 10.1186/s40662-019-0166-x

**Published:** 2020-01-07

**Authors:** Anna Pandolfi

**Affiliations:** Department of Civil and Environmental Engineering, Politecnico di Milano, Piazza Leonardo da Vinci 32, Milano, 20133 Italy

**Keywords:** Corneal biomechanics, Physiologic configuration, Tonometry modelling, Refractive surgery modelling

## Abstract

**Background:**

Biomechanics introduces numerous technologies to support clinical practice in ophthalmology, with the goal of improving surgical outcomes and to develop new advanced technologies with minimum impact on clinical training. Unfortunately, a few misconceptions on the way that computational methods should be applied to living tissues contributes to a lack of confidence towards computer-based approaches.

**Methods:**

Corneal biomechanics relies on sound theories of mechanics, including concepts of equilibrium, geometrical measurements, and complex material behaviors. The peculiarities of biological tissues require the consideration of multi-physics, typical of the eye environment, and to adopt customized geometrical models constructed on the basis of advanced optical imaging and in-vivo testing.

**Results:**

Patient-specific models are able to predict the outcomes of refractive surgery and to exploit the results of in-vivo test to characterize the material properties of the corneal tissue.

**Conclusions:**

Corneal biomechanics can become an important support to clinical practice, provided that methods are based on the actual multi-physics and use customized geometrical and mechanical models.

## Background

The eye is a multi-component biological structure. Each eye’s component has a conformation resulting from a microscopic organization (microstructure) related to its function within the assembly [1]. In this context, the spherical shape assumed by the cornea is due to the pressurization of the internal fluids, aqueous humor and vitreous humor [2]. Average values of the intraocular pressure (IOP) for healthy individuals is 15-18 mmHg, with small variations observed hourly, daily and weekly [3]. Higher IOP values are associated with grave pathological conditions such as glaucoma [4]. A robust homeostatic mechanism adjusts the aqueous humor outflow resistance to keep ocular pressures within relatively narrow acceptable bounds throughout most peoples’ lives [5]. Moreover, to preserve corneal transparency, other fundamental ion transport actions occur across the corneal endothelium.

All multi-physics regulation mechanisms observed in the cornea are the object of advanced studies. There is a convergence of opinion for some of them, while for others the central issues remain unclear and experimental results are contradictory [6].

In order to gain insight into the behaviour of the cornea, several mechanisms have been modelled separately by means of numerical models e. g., the remarkable examples given by models of cornea transport and swelling [7] and aqueous flow around IOLs [8, 9]. Computational mechanics has allowed for the simulation of the biomechanical responses of the eye to physiological actions [10], external actions [11–13], and geometric changes due to refractive surgery [14–17]. Interesting examples of applications on idealized geometries –able to qualitatively describe the mechanical response of portions of the eye’s anterior chamber under refractive correction– can be found in recent literature [18–24].

A numerical model is useful as long as it is able to capture the important physical characteristics of the system that the model wants to represent. If all the right physics are accounted for, although the model is verified only through a single particular experimental/theoretical comparison, it will be able to predict the behavior under different conditions.

The only way to achieve predictability is to include patient-specific geometrical features into the model and to account for all the phenomena that are of interest in the analysis. In the case of the cornea, whose main function is the refraction of light onto the retina, the geometry is of utmost importance. Additionally, the cornea has the important function of protecting the internal components of the eye, and thus it is characterized by a rather stiff and robust structure. Stiffness and robustness are conferred by a microstructure made of collagen fibrils organized in a precise architecture revealed by X-ray diffraction studies [25]. The collagen architecture – which provides various degrees of anisotropy dependently on the location – has strong implications on the biomechanics of the cornea.

In a complete model of the cornea, all the important mechanisms would be accounted for in order to evaluate realistically and quantitatively their interaction and to simulate the overall response to external actions and intervention.

Although a comprehensive virtual model of the anterior segment of the eye has not yet been realized, the examples mentioned in the previous paragraphs represent important steps towards the definition of a reliable numerical model of the cornea, although in most cases geometry and material properties were not associated with a particular patient. Interestingly, a few recent contributions have proposed combined experimental and numerical approaches to characterize the individual properties of the cornea [12, 13, 26, 27, 27–29], promoting an important advancement towards the construction of patient-specific models. These studies are characterized by the adoption of the state-of-the art computational modelling of soft tissues, and differentiate from other contemporary works based on excessively simplifying assumptions that hinder the predictability of the methods.

The following sections describe the features of the human cornea that, according to the experience and understanding of the writer, must be included in a predictive numerical model, especially if it is intended to be used in support of surgical interventions and therapeutical treatments.

## Methods

The realization of a numerical model of an organ, or of a portion of an organ, requires the consideration of all the physics involved in the processes that the model wants to reproduce. The refraction of the light primarily involves the cornea, the external and most powerful lens of the eye system. The cornea is a solid body with a proper shape but is rich in fluids and should be defined properly as a porous medium. Except for pathologic situations where the flux of fluids plays a primary role, in many applications of interest the cornea can be described as a dry solid and the action of the aqueous humor can be reproduced in terms of a uniform pressure. Obviously, this is true when the determination of the physiological steady distribution of strains (local dimensionless measures of changes of shape and volume) and stresses (local internal forces originating from the application of external actions to the body) is the main goal of the investigation, especially when the preoperative and postoperative configurations of the cornea in physiological conditions are compared.

In other cases, however, it is necessary to account also for the presence of the filling fluids because their mechanical interaction with the cornea affects the global distribution of strains and stresses and therefore modifies the macroscopic shape of the cornea. A typical example is the dynamic contactless tonometer (known as the air puff test) that causes an anomalous concave deformation of the cornea by applying a fast concentrated air jet to the center of the cornea. The interpretation of the test requires accounting for all the involved physics: dynamics of solids and fluids as well as interaction between solids and fluids.

**Fig. 1:**
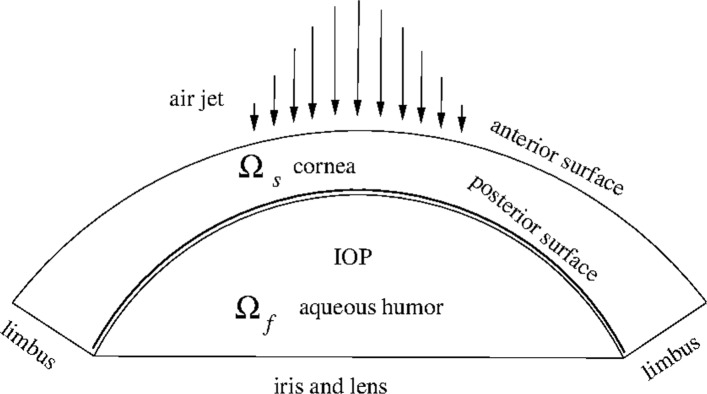
Schematic of the anterior chamber of the eye. The image is a meridian section of a 3D model, showing the solid domain of the cornea, with fixed boundary at the limbus and anterior surface where the pressure induced of an air jet can act, and the fluid domain of the aqueous, located between the cornea and the rigid iris and lens support. The interface between cornea and aqueous is in common between the two domains and, in dynamic conditions, represents a interaction interface between fluid and solid

All the aspects of the cornea have to be accounted for in the definition of a numerical model to be used in stress analysis: geometry, surrounding tissues, balance equations, loads, and materials. The corneal model described here refers to the schematic visualized in Fig. 1, showing the anterior chamber with solid cornea and the aqueous humor. In this model, the mechanics of the lens and the iris are disregarded and the two tissues are considered as rigid surfaces.

### Geometry

The geometry of the cornea is related to the refractive power and thus the use of the patient-specific shape becomes mandatory when the numerical model has to be used to simulate the changes of geometry induced by refractive surgery. Modern ophthalmologic instruments (corneal topographers and pachymeters) acquire the shape of the anterior and posterior surfaces of the cornea and of the anterior surface of iris and lens at a very high resolution, generally in terms of coordinates of clouds of points. These data can be manipulated in order to extract all the information on the local curvature of the cornea and to provide a map of the refractive power and optical aberration of each cornea.

**Fig. 2:**
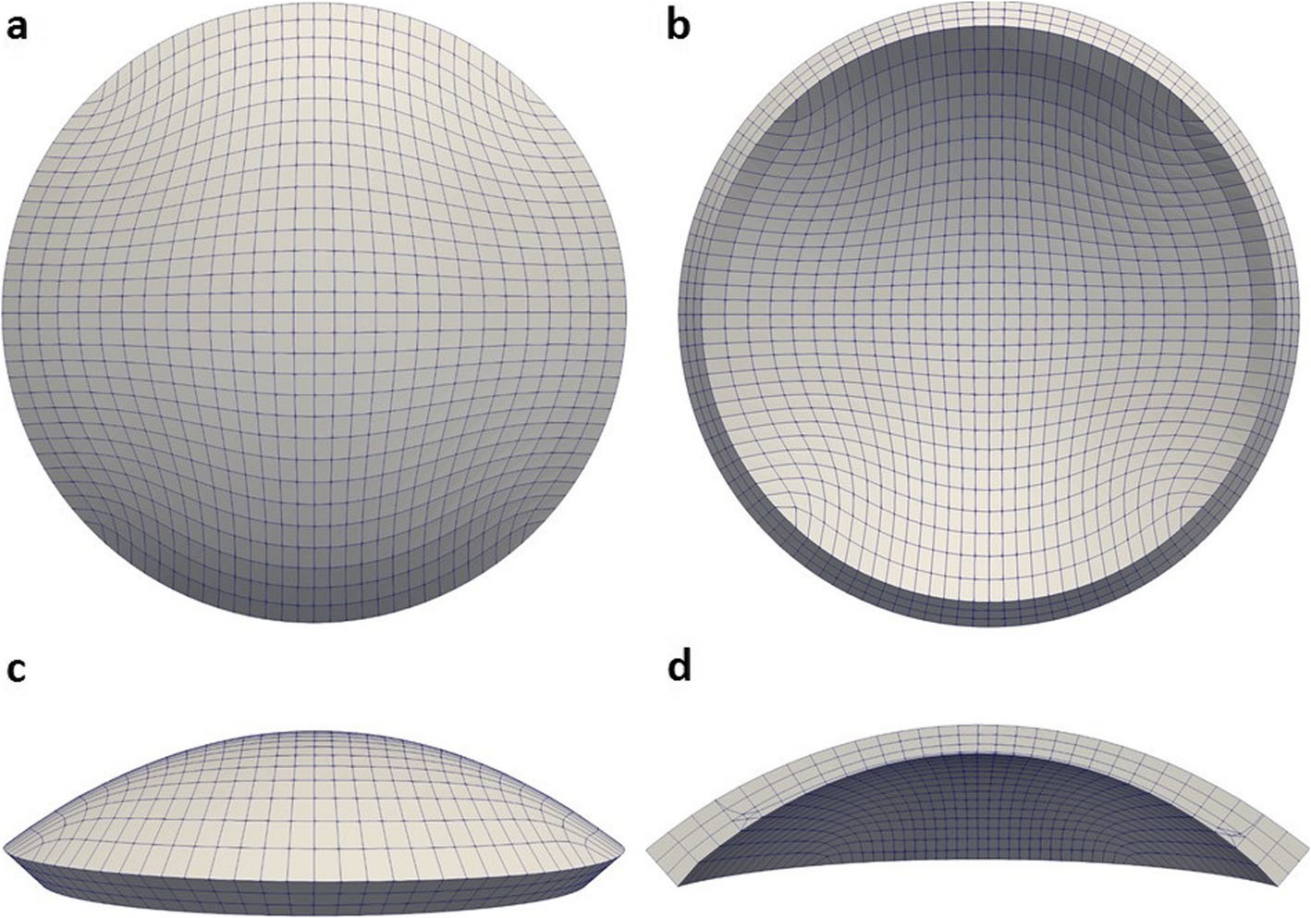
Example of a finite element mesh for a human cornea. The solid model is derived from patient-specific geometries acquired by means of an advanced corneal topographer. Once the discretization level (mesh size) has been decided, as dictated by the particular application, the coordinates of the nodes lying on the anterior and posterior surface are determined by interpolation over the grid of topographer points. **a** Anterior view. **b** Posterior view. **c** Side view. **d** Meridional nasal-temporal section

**Cornea.** The set of points can be transferred to a solid modeler to create a full three-dimensional geometrical model of the cornea, and, according to the desired numerical application, the model can be discretized in small portions with a predefined simple shape (either hexahedra or tetrahedra) in view of use in finite element codes [11, 16, 30]. An example of a finite element mesh of a patient-specific solid model of the cornea, made of 8-noded exahedra, is shown in Fig. 2. In this case, the discretization has been designed in order to include in a smooth way the architecture of the collagen fibrils that reinforce the stroma. The level of the discretization is parametrized in terms of number of subdivisions in the in-plane projection and across the thickness. In previous works [10, 30], we carried out convergence analyses that demonstrated that a number of elements between three (for static applications) and five (for dynamic applications) across the thickness are sufficient to describe the distribution of the stress with an accuracy (difference of less than %) requested by biomechanical applications.

**Fig. 3:**
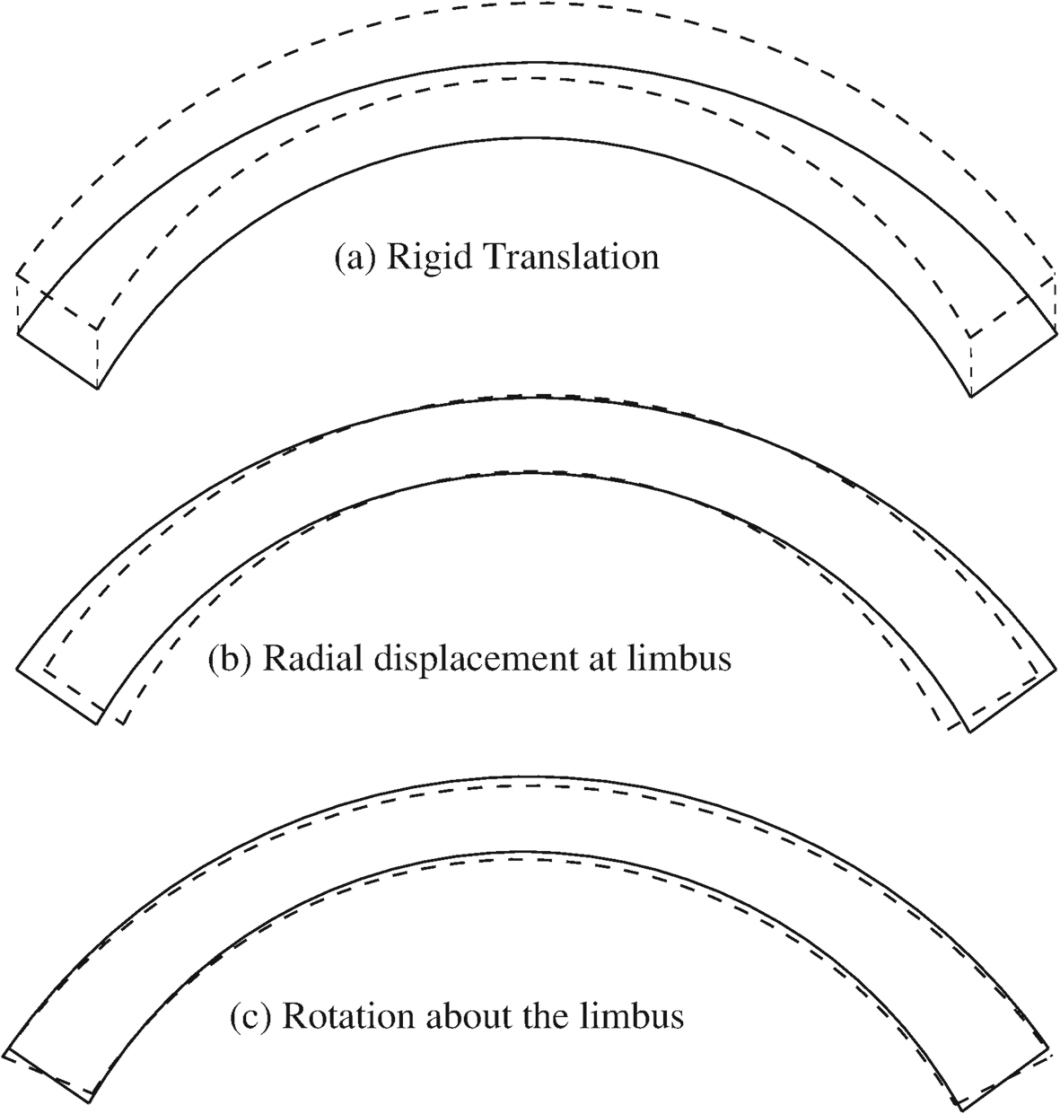
Motion and constraint of the limbus. **a** The deformation of the axis-symmetric sclera leads to a uniform displacement of the cornea in the direction of the optical axis, which does not induce deformations. **b** The stiff limbus does not extend circumferentially neither radially. **c** The only motion compatible with the stiff limbus located between more compliant tissues, cornea and sclera) is a rotation about its circumferential midline

**Limbus and surrounding tissues.** In many corneal models, the tissues surrounding the cornea, in particular limbus, iris and sclera, are excluded. The reason for excluding these tissues is related to the impossibility of knowing their mechanical properties because, unlike the cornea, they are not directly accessible for mechanical examination. The inclusion of additional tissues will introduce into the model uncertainties that are not easily quantifiable, with unpredictable consequences on the results. Nevertheless, the choice to exclude from the model portion of tissues is counterbalanced by the need to define a suitable support for the limbus. As explained in [18], the strain and stress state of any tissue are dependent only on relative displacements between close points. A uniform displacement does not cause deformation, but only a change of configuration, and it is denoted as a rigid body motion. The effect of most of the surrounding tissues is to cause a rigid body motion to the cornea (e. g., the effect of the ocular muscles). Given the dominant axis-symmetry of the eye, the deformations of the sclera must lead to a uniform displacement of the cornea in the direction of the optical axis, Fig. 3(a). Moreover, the limbus is a rather thick and rigid structure, and under the action of the IOP it makes sense to assume that the limbus does not extend circumferentially, causing a change of the inplane diameter of the cornea, Fig. 3(b). Therefore, the only motion that can induce a change of shape of the cornea is a moderate rotation of the limbus around its axis, see Fig. 3(c), caused by the adjacency of a very stiff structure (limbus) with more compliant structures (cornea and sclera). We observe that the geometry of the cornea favors the deflection and the bending of the central portion, while the rotation of the limbus remains certainly less evident.

**Fig. 4:**
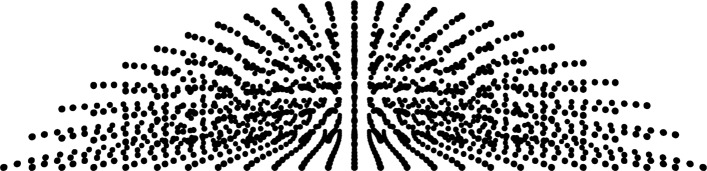
Example of a meshfree discretization of the aqueous. The fluid model is obtained by filling with particles the patient-specific geometry of a posterior cornea. Once the discretization of the solid model has been assigned, the coordinates of the particles within the anterior chamber are obtained through a regular subdivision of the volume

**Aqueous.** To model the extended deformations observed in fluids, finite elements are less practical than alternative CFD approaches, such as finite volumes, particle methods, or various meshfree methods. A promising approach uses a lagrangian meshfree discretization of the fluid, for example with the modified fluid particle meshfree (MFPM) method, which was recently proposed for a three-dimensional model of the air puff test [13]. The discretization of the fluid must conform to the one of the solid to guarantee the same accuracy to the solution of the solid and of the fluid parts (Fig. 4).

### Governing equations

The simulation of biomechanical problems involving the cornea requires the solution of the equations that govern the motion of solids and fluids. The conditions of static or dynamic equilibrium in deformable solids and flowing fluids are expressed by means of well-known differential (i.e., that include space and time derivatives) equations that generalize Newton’s laws of mechanics by introducing the concept of internal engagement or stress and accounting the change of shape and volume of the bodies by means of strains. While the strains are exclusively related to geometrical changes and are measurable, stresses can only be estimated on the basis of balance conditions that account for the applied external actions.

The mechanics of the cornea is governed by the dynamic equilibrium equation

1 $$ \nabla \cdot {\mathbf{P}} + {\mathbf{B}} = \rho_{c} \ddot {\mathbf{U}}_{c}  $$

where **P** is the stress tensor, **B** the force per unit of volume, **U**_*c*_ the displacement vector, *ρ*_*c*_ the cornea density, ∇· the divergence operator, and a superposed dot the derivative with respect to time *t*. The differential equation holds over the volume of the cornea and must be solved by considering initial conditions, in terms of displacement and velocity fields, and boundary conditions, in terms of assigned displacements at the limbus, of an eventual pressure history on the anterior surface, and the interaction with fluids on the posterior surface.

The mechanics of the aqueous humor is governed by the continuity equation

2 $$ \frac{D \rho_{f}}{D t} + \rho_{f} \nabla \cdot {\mathbf{V}}_{f} = 0 \,,  $$

where *ρ*_*f*_ denotes the fluid density, **V**_*f*_ is the fluid velocity, *D*/*Dt* the time derivative, and by the dynamic equilibrium equation,

3 $$ \rho_{f} \frac{D {\mathbf{V}}_{f}}{D t} = - \nabla p_{f} + \nabla \cdot \mathbf{\boldsymbol{\tau}} \,,  $$

where *ρ*_*f*_ denotes the fluid density, *p*_*f*_ the fluid pressure, ***τ*** the deviatoric stress tensor, ∇ the gradient operator, and ∇· the divergence operator. Both the differential equations, defined over the volume occupied by the fluid, have to be solved by considering the initial conditions, in terms of velocity field, and boundary conditions, that include zero flux (by assumption) across lens and iris, and the knowledge of the interaction with the solid on the interface with the cornea.

In dynamics, the boundary conditions on the interface between the cornea and the aqueous should be framed under a fluid-solid-interaction (FSI) problem. The velocities of the moving cornea are transmitted to the fluid on the posterior surface of the cornea. In turn, the motion of the fluid causes modifications in the distribution of the fluid pressure which in general will be non-uniform at the cornea-aqueous interface.

Under quasi-static conditions, however, the velocity of the fluid is zero, the pressure of the fluid at the interface is constant, and it is not necessary to solve the equation of the fluid.

In the present implementation of the corneal model, the iris and the lens are modelled as rigid surfaces, given the impossibility to obtain the correct material properties for these tissues.

### Materials

The cornea is a typical biological tissue, characterized by a large deformability and progressive stiffening. It reveals a certain degree of incompressibility (no volumetric variation under load) and a strong dependence on the direction of loading (anisotropy). In physiological conditions the cornea is stressed by the IOP.

The mathematical description of the behavior of a material is called constitutive law. A constitutive law relates the strains to the stresses. A constitutive law, in general, must establish the suitable relations between all the components of a strain tensor and the all the components of a stress tensor.

**Cornea.** The organization of the corneal tissue is complex, but from the mechanical point of view the important aspects are related to the collagen, the structural component of the stroma. The collagen is organized hierarchically in fibrils and lamellae following a complex architecture that has been discovered more than three decades ago [25, 31].

In the central area of the cornea, the lamellae are preferentially oriented in two directions: nasal-temporal (NT) and superior-inferior (SI). This organization involves about 60% of the fibrils, while the remaining 40% are randomly oriented [32]. The change in curvature in the limbus zone is related to the presence of a consistent amount of fibrils aligned in the circumferential direction. The distribution of the fibrils is not homogeneous across the corneal thickness. Biomedical imaging has revealed recently that collagen lamellae in the posterior cornea are commonly twice as thick as those in the anterior [33] and interlamellar interaction results from interweaving [34], leading to a shear stiffness three times larger in the anterior third of the stroma than the one in the posterior third of the stroma. At the limbus, the larger stiffness is shown at the posterior side, where the limbus merges with the iris. The variability of the characteristics of the collagen across the stroma thickness is not apparently related to a particular mechanical performance in physiological conditions, but may have implications during the execution of mechanical tests [11].

**Fig. 5:**
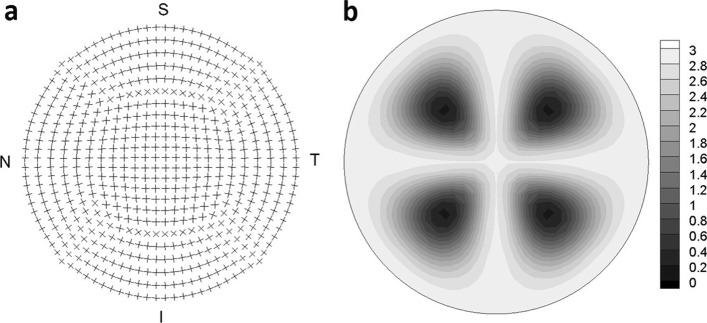
Fibril orientation and distribution level. All collagen of the stroma is organized in microstructures called lamellae, that run rather parallel to the mid surface of the cornea. Almost 60% of the resulting collagen fibrils distribution are uniformly dispersed, leading to an isotropic behavior. The remaining 40% is instead oriented in specific directions. **a** According to X-ray diffraction findings, at the center of the cornea collagen is organized in an orthogonal configuration, and at the limbus it runs circumferentially, although it is requested the presence of radial fibrils to guarantee the integrity of the body. **b** Maps of the coefficient *b* that defines the level of the anisotropy of 40% of the stromal fibrils. A small value of *b* corresponds to an isotropic distribution, a large value *b*>2 correspond to a marked anisotropy

A schematic representation of the collagen architecture in the stroma has been proposed in [10], see Fig. 5. The fibril organization has been designed in order to fit the particular finite element discretization visualized in Fig. 2. The orientation of the fibrils, indeed, is built in the mesh generation and it follows the orientation of the edges of the finite element hexahedra, Fig. 5(a). Since the fibrils in the cornea are not oriented isotropically ubiquitously but, in precise locations, they show preferential directions, the variability of the spatial distribution of the fibril orientation may be assigned through the axisymmetric von Mises distribution defined over the unit sphere as:

4 $$ \rho \left(\Theta\right) = \frac{1}{2 \pi I} \exp\left(b\cos{2\Theta}\right),  $$

where *Θ* is an angle spanning over a meridian of the sphere, *I* is the normalization coefficient

5 $$ I = \frac{1}{\pi}\int_{0}^{\pi} { \exp\left(b\cos{2\Theta}\right)d\Theta}  $$

and *b* is the concentration parameter that accounts for the dispersion of the distribution. The parameter varies from *b*=0, denoting a perfectly isotropic distribution, to *b*=2.8 denoting a rather strong orientation as the one observed at the corneal center and at the limbus, see Fig. 5(b).

Upon loading in the physiological range, the cornea manifests a reversible behavior, therefore it can be treated as a hyperelastic material. Hyperelasticity is a very convenient approach to deal with reversibility. The idea is that a deformed system accumulates energy that is fully recovered when the cause of the deformation is removed. The advantage implied by hyperelasticity is the possibility to describe the full behavior of the material through a unique scalar strain energy density function *Ψ*, dependent on the nine components of the strain. The knowledge of the scalar strain function is the sole requirement for obtaining the stress from the given strain.

Although several material models have been proposed, the most used models assume the decomposition of the strain energy density of the material in the sum of volumetric *Ψ*_v_, isochoric isotropic *Ψ*_i_, and anisotropic *Ψ*_a_ parts [35]. This choice, accompanied with a separation of the arguments of the three parts, leads to a noteworthy simplification of the mathematical model and avoids numerical issues connected to incompressibility. The strain energy density is expressed as

6 $$ {\Psi} = \Psi_{\text{vol}}(J) + \Psi_{\text{iso}}(\overline I_{1}, \overline I_{2}) + \Psi_{\text{aniso}} ({I^{*}_{4}{M}}, \boldsymbol{\sigma}_{M}).  $$

The term *Ψ*_vol_ has to be considered as a penalty term to enforce the material incompressibility. The term *Ψ*_iso_ describes the behavior of the isotropic underline proteoglycan matrix and of the 40% portion of randomly distributed fibrils and is assumed to be dependent on two scalars only, $\overline I_{1}$ and $\overline I_{2}$, connected to a suitable measure of strain (i.e., the isochoric Cauchy-Green deformation tensor). The term *Ψ*_a_ addresses the anisotropic contribution two non-randomly oriented collagen fibril families. For a set of fibrils strongly aligned in the direction **a**, anisotropy is included through isochoric scalars $I^{*}_{4}{M}, \boldsymbol {\sigma }_{M}$ (average and variance related measures of the distribution) of the main orientation of the fibrils **a** [10, 23, 24, 35]. Although compressed fibrils may have a very reduced stiffness related to local buckling [36, 37], the material model used in this study does not exclude the contribution of compressed fibrils. The actual role of compressed fibrils has initiated an interesting discussion concerning a criterion to switch between tension and compression in fiber reinforced material models [38, 39]. Although we believe that this criterion would be fundamental in materials made only by fibers, in our model we do not account for it because the particular structure of the cornea, made of collagen fibrils immersed into a matrix of elastin and proteoglycans, is able to provide some confinement to the compressed fibrils ruling out the possibility to observe local buckling. This point is still open, and perhaps our choice is not an ideal one. Nevertheless, we have observed that, in spite of considered compressed fibrils, our models seem to be sufficiently predictive in all applications. The particular forms of the strain energy density have to be chosen according to available experimental data, possibly obtained through in-vivo tests on human corneas. The expressions used in this study, that have been developed and verified in [35], are recalled in Appendix A.

Given the nature of the applications considered here, reproducing physiological states or very fast dynamic tests, where delayed or viscous behaviors play no role, we disregard the viscosity of the cornea, observable and measurable only in slow relaxation tests on excised strips [40].

**Aqueous.** The deviatoric stress is related to the fluid velocity through the Stokes’ constitutive relation as

7 $$ \boldsymbol{\tau} = 2 \mu_{f} \, \text{sym} \nabla V_{f} \,,  $$

where *μ*_*f*_ is the fluid viscosity, while the constitutive relation for the fluid pressure can be taken in the form of the Tait’s equation of state, cf. [41],

8 $$  p_{f} = p_{0} + \rho_{0} \, \frac{c^{2}}{\gamma} \left[ \left({\frac{\rho_{f}}{\rho_{0}}} \right)^{\gamma} - 1 \right] \,,  $$

where *p*_0_ and *ρ*_0_ are the reference pressure and density, respectively, *c* a parameter related to the speed of sound in the fluid, and *γ* a material parameter, which reasonably for water can be assumed to be *γ*=7 [42].

When a problem concerning fluid-solid interaction has to be solved, a good approach consists in combining the finite element discretization of the solid with a meshfree discretization of the fluid. Recently, we have developed a partitioned code that solves separately the equations of motion for solid and fluid and enforces the interaction boundary conditions alternatively on the solid-fluid interface [12, 13].

### Unstressed geometry

An important feature of codes that analyze the stress state of highly deformable bodies is the recovery of the unstressed configuration. In-vivo imaging provides the deformed geometry of the cornea in a stressed state that balances the physiological IOP. The stress state is unknown and is occasionally referred to as pre-stress. The correct simulation of the mechanical response of the cornea requires the knowledge of either the pre-stress state or of the unstressed geometry to which the IOP must be applied. In our work, we choose to recover the unstressed geometry, in contrast to a few alternative approaches that have chosen to identify the pre-stress state [20]. The physiological configuration is used to define the target discretization with coordinates **X**_0_. The recovery procedure requires the execution of a sequence of static analyses under the physiological IOP. The first analysis sets the coordinates **X**^1^=**X**_0_. At the iteration *k*, the static analysis furnishes the displacements **U**^*k*^, used to estimate the new trial coordinates **X**^*k*+1^=**X**_0_−**U**^*k*^. The procedure ends when the magnitude of the difference between two sequential coordinates becomes smaller than a predefined tolerance [10].

## Results

Finite elements can be used to model several mechanical problems relevant to the human cornea. In the following examples, we make use of the material model reported in Appendix A, while the adopted material properties are listed in Table 1. The results presented here have been obtained by using the imaged geometry of ten corneas chosen in a random way from a large set of informed patients that underwent refractive surgery. Images used in this work were collected by the same experienced surgeon using a high definition corneal tomographer coupled with a pachymeter, according to a protocol approved by the Italian Data Protection Authority and to the principles expressed in the Declaration of Helsinki. Purely geometrical data were anonymized and de-identified prior to the transmission to the authors and disjoined by all the other clinical information (age, gender, ethnicity) and, in particular, by the IOP. In all numerical simulations, the physiological IOP is assumed to be 16 mmHg.

**Table 1:** Material parameters used in the numerical applications

*K* [MPa]	*μ*_1_ [MPa]	*μ*_2_ [MPa]	*k*_1 *M*_ [MPa]	*k*_2 *M*_	*b*_*M*_
5.5	0.08	−0.02	0.05	200	[0.2, 2.8]

**Refractive surgery.** The model can be used to estimate the change of corneal shape, refractive power, and stress distribution induced by laser ablation refractive surgery. Our simulations aimed at estimating the actual distribution of stress and strains within the cornea before and after the surgery, and to provide an indication of the increment of the engagement of the material, which is the main interest for a surgeon because in the long term it can lead to material instabilities. Needless to say, the model should account for the physiological, patient-specific value of the IOP.

The refractive surgery that can be modelled more easily with finite elements is the photorefractive-keratectomy (PRK). The surgery removes a thin portion of the anterior cornea, including the epithelium, the Bowman’s membrane, and a portion of the stroma. The design of the ablation is suitably chosen so that, after healing and re-epithelization of the anterior surface, the cornea shape will provide the desired refractive power.

As far as the numerical modelling is concerned, the FEM approach allows the use of several materials, and thus each element can be characterized by different material properties according to its position within the discretization. The overall stiffness of the cornea is the result of the assembling of the behavior of all the elements. To model the corneal ablation, we thin the elements on the anterior layer preserving the local distribution of the material [11, 16, 17]. The code provides the distribution of stress and strain before and after the surgery by considering the pre- and post-operative geometry acquired with imaging. Furthermore, the code is equipped with a reprofiling algorithm that, based only on the surgical design of the ablation i.e., without the need of knowing the post-operative configuration of the cornea, is able to provide a distribution of strains and stresses [16].

**Fig. 6:**
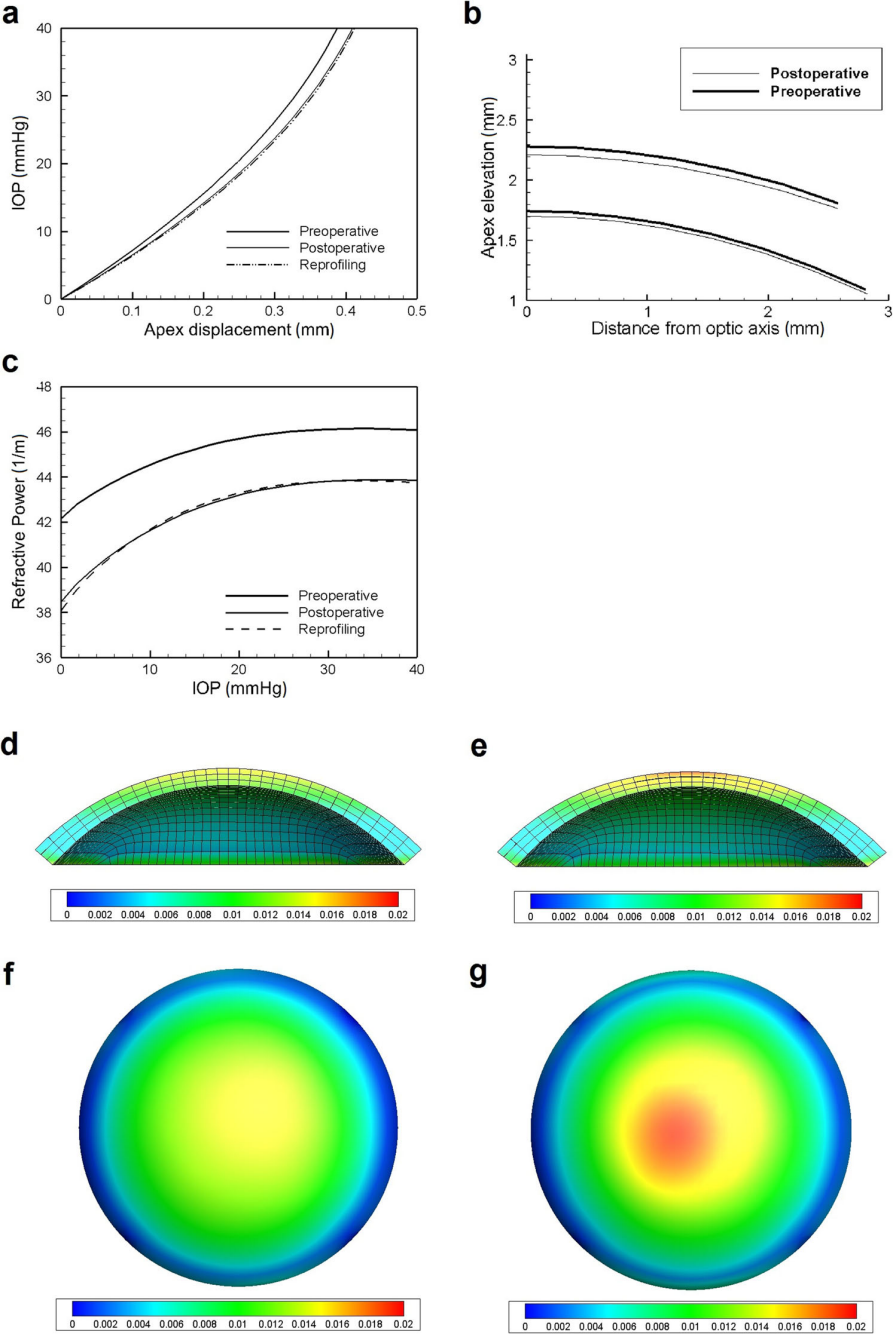
Modelling of PRK refractive surgery. Simulation, in a patient-specific geometry, of a PRK refractive surgery. **a** Comparison between the pre-operative and postoperative curve IOP versus apex-displacement. The plot shows the curve obtained using the preoperative patient-specific geometry, the postoperative patient-specific geometry (both obtained using data from imaging), and the prediction of a reprofiling procedure implemented in the code. **b** Comparison of the preoperative and postoperative profiles of the patient-specific cornea. **c** Refractive power of the cornea as a function of the IOP as estimated by the code on the basis of the geometries reconstructed from imaging before and after PRK surgery. The figure shows also the prediction of the reprofiling procedure embedded in the code. **d** Distribution along the NT direction meridian of the horizontal component of the stress in the preoperative configuration. **e** Distribution along the NT direction meridian of the horizontal component of the stress in the postoperative configuration. **f** Anterior surface distribution of the horizontal component of the stress in the preoperative configuration. **g** Anterior surface distribution of the horizontal component of the stress in the postoperative configuration

An example of reprofiling with PRK is shown in Fig. 6, where the preoperative and postoperative configurations of a patient-specific cornea are compared. The plot in Fig. 6(a) shows the IOP versus apex displacement curves obtained using the preoperative patient-specific geometry, the postoperative patient-specific geometry, and the prediction of the reprofiling procedure implemented in the code. Figure 6(b) compares the preoperative and postoperative profiles of the cornea in the central optic zone of 3 mm. Figure 6(c) shows the evolution of the refractive power along the NT meridian as a function of the increasing IOP for the models of the preoperative and postoperative corneas as obtained from imaging. The figure also shows, in the dashed line, the prediction of the reprofiling algorithm. Figures 6(d) and (e) show the distributions along the NT direction meridian of the horizontal component of the stress in the preoperative and postoperative configurations, respectively. Figures 6(f) and (g) show the in-plane map of the distributions of the horizontal component of the stress in the preoperative and postoperative configurations, respectively, visualized over the anterior surface of the cornea. The predictions of the reprofiling procedure have been compared with the actual post-operative outcomes, showing a very good correspondence, indicating a validation of the model.

**Quasi-static indentation test.** The model can also be used to simulate the indentation with a probe, a tool derived from the contact tonometer that can be used to assess the stiffness of the cornea and to identify the material properties of the chosen material model. The action of an opto-mechanical testing device applied at the corneal apex is modeled numerically in terms of a displacement history imposed to the nodes in contact with the mechanical probe. The probe, a 0.5 mm diameter cylindrical indenter with a hemispherical tip [43], advances into the corneal apex up to 600 *μ*m to create a small concavity in the cornea in 60 steps. The action of the probe test is simulated after applying the physiological IOP to the cornea.

**Fig. 7:**
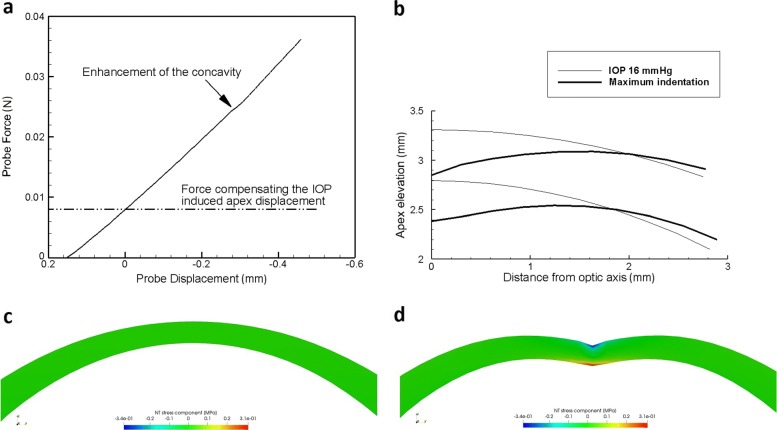
Modelling the probe indentation test. Numerical simulation of the probe test. **a** Force versus apex displacement curve. **b** Cornea profile along the NT direction at the maximum displacement of the probe, corresponding to 0.6 mm indentation of the cornea. Two thin lines refer to the anterior and posterior surfaces of the cornea at the physiological IOP = 16 mmHg. **c** Distribution of the NT stress component along the NT meridian section at the physiological state. **d** Distribution of the NT stress component along the NT meridian section at the maximum indentation state

The results of the simulations of quasi-static contact tests are reported in Fig. 7. Figure 7(a) shows the global mechanical response in terms of probe force versus probe displacement, identical to the apex displacement. At the beginning of the test, the probe recovers the displacement induced by the IOP. When the apex reaches the position on the optical axis corresponding to the unstressed configuration, the force exerted by the probe balances the distribution of the physiological IOP. The stress distribution, however, is non zero since the configuration is different form the unstressed configuration. The anterior surface follows the shape of the probe, and the displacement becomes negative (i.e., the apex locates at a position inferior to the one in the unstressed configuration) with the creation of a concavity, see Fig. 7(b). At about 0.4 mm displacement of the probe, −0.3 mm of indentation, a change in the slope of the curve, corresponding to a sort of enhancement of the concave configuration (like a snap-through), is observed. The mechanical response to the probe action induces a modification of the stress distribution. The NT stress component along the NT meridian is visualized at the beginning of the probe test, Fig. 7(c), and at the maximum deflection of the cornea, Fig. 7(d). In the latter condition, the model predicts a stress reversal, with tensile stress at the posterior side of the cornea and compressive stress at the probe contact point. Note that, during the probe stress, the shear stress components remain one or two orders of magnitude smaller than the normal stress components. A comparison of the test between different material models can be found in [11].

**Dynamic contactless test.** The combined model cornea-aqueous is used to simulate dynamic tests, such as the air puff test. The action of a contactless ocular tonometer, that uses a localized air jet to induce the oscillation of the cornea, is modelled by applying an estimated pressure history on a small area of the central anterior cornea. The sudden pulse exerted by the instrument causes the inward motion of the cornea, which transits through an applanation, and successively snaps into a concavity. When the air pulse pressure ceases, the corneal tissue recovers the original configuration, transiting through a second applanation. Since the actual space and time profile of the air jet pressure are unavailable, the imprint of the air jet has been estimated, through several parametric analyses, using simplified analytical expressions [30], see Appendix B.

**Fig. 8:**
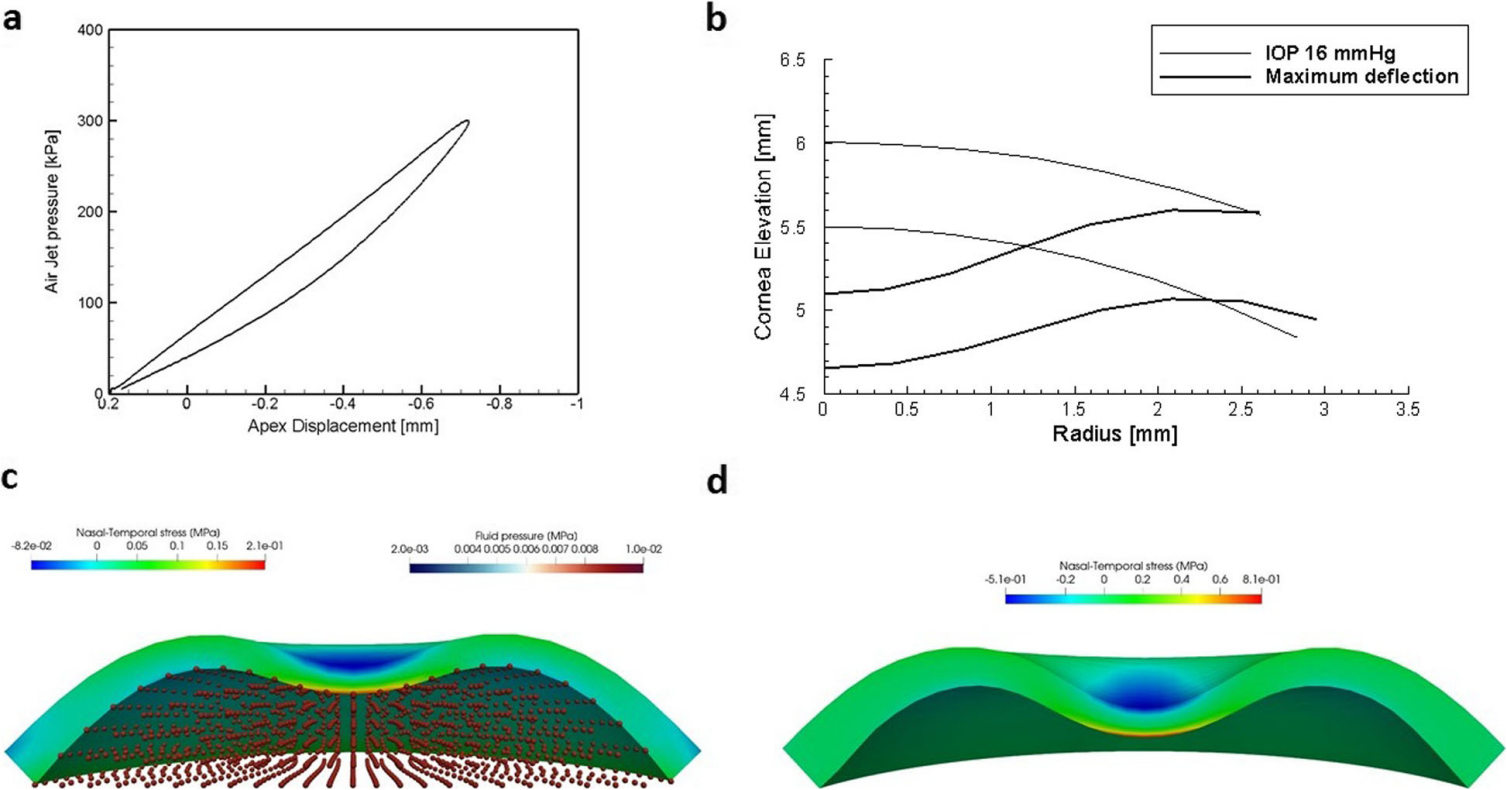
Modelling the dynamic contactless tonometer test. Numerical simulation of the contactless tonometer test. **a** Air jet pressure versus apex displacement curve. **b** Cornea NT profile at the maximum value of the air jet. Two thin lines visualize the anterior and posterior surfaces of the cornea at the physiological IOP. **c** Distribution of the NT stress component along the NT meridian section at the maximum deflection of the cornea (correct physics). **d** Distribution of the NT stress component along the NT meridian section at the maximum deflection of the cornea in the absence of the fluid (wrong physics)

Selected results of the simulations of dynamic contactless tests are visualized in Fig. 8. Figure 8(a) shows the mechanical response in terms of air jet pressure versus apex displacement. Figure 8(b) shows the profile of the cornea corresponding to maximum value of the air jet pressure. Figure 8(c) shows the stress in the cornea and the distribution of the pressure in the fluid in correspondence to the maximum value of the air jet pressure. The interaction algorithm between cornea and aqueous has been described in [12], where a simplified isotropic material model without fibrils has been used for the cornea.

For the sake of comparison, Fig. 8(d) shows the stress distribution in the cornea when the presence of the aqueous is not taken into account. The deflection of the cornea is too large because of the missing contrast of the fluid. A detailed description of the model used in this simplified simulation can be found in [27, 30].

## Discussion

The examples of application of numerical technologies (finite element and meshless discretizations) to the biomechanics of the cornea reveal the potential of numerical methods, that can become a valid support to refractive surgery and therapeutic treatments.

In the simulations presented here, the finite element discretization has been designed with the precise aim of capturing the main orientation of the collagen fibril. The smooth transition from an orthogonal orientation NT and SI at the center of the cornea to an alternative orthogonal orientation, circumferential and radial, at the limbus reflects the architecture of the collagen organization, which cannot be abrupt, to guarantee the continuity of the refractive properties of the tissue. The design has proven to be particularly useful for the definition of the orientation of the fibrils (since the main direction of the anisotropic fibers lie parallel to the edges of the elements) and for the visualization of the output of the calculation, since the NT and SI meridian are implicitly included in the design and all information on these meridians are easily achieved. Many alternative types of discretization can be found in the literature, justified by other ways of reasoning than the present one. In general, alternative discretizations are characterized by a very fine size of the mesh in the central cornea, which leads to a much larger number of elements with respects to the present models. Clearly, refined models may be more accurate (provided that a sufficiently precise material model is adopted). However, an exceedingly fine discretization may imply very long computational times, not compatible with the normal times of a medical examination and if a quick response expected in real time applications.

Another observation concerns the modelling of the tissues surrounding the cornea. While the cornea has been sufficiently characterized from the mechanical point of view, the other tissues of the eye (limbus, sclera, iris, lens and so on), too small or too thin to be tested in-vivo with the present technology, did receive scant attention till now. The result is that such materials are not sufficiently well known and, moreover, it is not easy to define patient-specific material properties that can be used in a whole eye model. The exclusion of the surrounding tissues is indeed a limit in a cornea model, but, from the engineering point of view, it is not too difficult to find an equivalent constraint at the limbus able to reproduce the overall behavior of the missing tissues without the need of modelling each of them. As far as this point is concerned, alternative points of view can be found in the literature. In some cases, the sclera is included in the model of the eye, regrettably disregarding the other stiff structures at the separation between cornea and sclera (iris, lens, etc).

The choice of the material model is fundamental in the prediction of the stress distribution. The knowledge of the stress is of utmost importance in order to detect zones of the tissues that can undergo degeneration or damage, leading to undesired phenomena of excessive deformation (see the case of post-LASIK ectasia). The distribution of the stresses in a body is dependent on the material model used to describe the behavior of the material. The stress is a quantity that cannot really be measured, but it can be only assumed on the basis of the observable quantities that are related to the changes of geometry. However, the effect of the stress (viscous and time dependent behaviors) are well known to ophthalmologists. An excess of stress, often related to an excessive therapeutical thinning of the cornea, is the main reason for undesired phenomena like localized ectasia. The material model adopted to describe the behavior of the stroma must be as faithful as possible to the real characteristics of the tissue. Anisotropy induced by the presence of collagen fibrils is a fundamental ingredient, although a specific microstructural description of the collagen architecture might be the best avenue to pursue in order to be able to model diseases such as keratoconus [44].

Another important aspect of numerical modelling is that surgery that does not require the incision of a tissue is much easier to simulate. In fact, any new surface created in the cornea requires to be explicitly modelled even in the original preoperative geometry, in order to compare with accuracy the changes induced by the remodelling of the tissue. Thus, modelling PRK surgery is rather easy since it requires the change of the coordinates of the sole anterior surface. Contrariwise, modelling LASIK, LASEK and SMILE is computationally more complicated. The incision made to create the flap is a physical interface within the tissue and requires the use of frictional contact to model the postoperative condition. The final configuration of a post-PRK and post-LASIK surgery may be very similar, but the internal distribution of the stresses would be very different.

As many times remarked in our previous works [12], the biomechanical and optical behavior of a patient-specific cornea can be predicted by means of a numerical approach only when patient-specific geometry, material properties and IOP are assigned. Unfortunately, only the geometry can be easily accounted for, while patient-specific material properties and IOP require some important and demanding work to be correctly inserted into the model. The estimation of material properties and IOP can be done with techniques proper of inverse analysis. Inverse analysis consists of assigning the geometry and the material models of a system, and in finding the optimal material parameters by means of a sequence of stress analyses. The value of the parameters used in each analysis of the sequence are selected on the basis of an error computed between the displacement of the system as estimated by the numerical model and the same displacements measured in experiments. To provide realistic values of the material properties, the comparison between numerical predictions and experimental results must be done on in-vivo tests. The two most interesting tests are the ones that locally modify the shape of the cornea: the probe indentation tests and the dynamic contactless tonometer. While for the probe test no particular care has to be taken, since the test is very slow and conducted in quasi-static condition, the numerical model of the air puff test cannot disregard the presence of the fluid. As a matter of fact, an inverse analysis based on the air puff test conducted without fluid will overestimate the material properties of the cornea, leading to a completely unreliable model, not useful for any clinical application.

It is important to observe that the imprint of the air puff test as exerted by any commercial device is not available (no factory will ever provide this confidential information), therefore, at present, the test cannot be used to identify material properties of the cornea. It is also important to remark that an anisotropic tissue requires more than one test to be characterized.

One of the aspects that can be elucidated with numerical simulation, but which has not been explored here, is the apparent reduction of the IOP following refractive surgery. The minor reading offered by the tonometer is the consequence of the calibration of the instrument made with reference to the average corneal thickness. The reading of a tonometer is obtained when the cornea flattens under the pressure exerted by the tonometer tip. Mechanically, flattening is the result of the force exerted by the IOP and of the stiffness of the corneal tissue, which depends on the corneal material and thickness. It is clear that, after surgery, the stiffness of the cornea reduces because the thickness reduces, therefore the force necessary for flattening the cornea is lower. A numerical model capable of reducing the thickness of the cornea would allow modelling the flattening of the cornea caused by the action of the tonometer. The force exerted by the tonometer tip would be an automatic outcome of the calculation, and it will reduce with the cornea thickness at the same IOP. Thus, a numerical model would be able to explain the somehow inexplicable reduction of the postoperative IOP readings.

A final comment concerns the presence of compressive stresses in the cornea during the execution of the probe test and of the air puff test. Many authors disregard the contribution of the fibrils in a compressive state, since thin filaments are very good in carrying tensile stresses, but they become unstable under compressive stresses. Instability induces a change of configuration and the redistribution of the stress on the surrounding tissues. Although this concept is well known, the behavior of compressed fibrils when immersed in a matrix that is able to provide some confinement (thus to offer some support to the change of the configuration) may not be exactly the same when compared with unconfined compressed filaments. Therefore, the exclusion of compressed fibrils when analysing the cornea should not be taken as an unavoidable necessity, but should be verified using inverse analysis. Moreover, the numerical procedures that must be activated in order to exclude compressed fibrils in a spatial distribution are rather complicated and uncertain, and may spoil the hypothetical advantages of using a purely tensile fibril model [38].

## Conclusions

This work is a demonstrative study to show the potential and versatility of numerical models of the cornea. Numerical applications are already available, and patient-specific geometries are easily obtained through advanced 3D imaging. The numerical model can provide information on the effective post-operative shape of the cornea, and the corresponding map of the refractive power. What is still missing is an experimental in-vivo protocol that, by a combination of non-invasive mechanical tests, is able to feed the numerical model with the exact (i.e., patient-specific) material properties and the exact IOP. Patient-specific models would not need nomograms or searches within million cases to find the closest similarities, because they will predict the mechanical outcome of a surgery as the answer of a body to mechanical actions performed on it.

## Appendix A

The material model adopted here for the stroma accounts for the presence of reinforcing collagen fibrils statistically distributed into a matrix of elastin and proteoglycans. The collagen fibers follow a statistic probability density function, according to a second order approximation [35]. In Eq. (6), the term *Ψ*_v_ is regarded as a penalty term to enforce weakly the incompressibility constraint and assumes the operative form. *Ψ*_v_ depends on the jacobian *J*= det**F**, where **F**=*∂***x**/*∂***X** is the deformation gradient as

$$ \Psi_{\text{vol}}(J) = \frac{1}{4}\, K \,(J^{2} - 1 -2 \log{J}) \,, $$

where the coefficient *K* corresponds to a volumetric stiffness coefficient, related to the bulk modulus. The term *Ψ*_iso_ describes the behavior of the isotropic components of the stroma material, including the elastin and the proteoglycan composing the matrix and the 60% portion of fully dispersed fibrils. The term is modelled according to Mooney-Rivlin’s strain energy function

$$ \Psi_{\text{iso}}(\overline I_{1}, \overline I_{2}) = \frac{1}{2} \mu_{1} (\overline{I}_{1} -3) + \frac{1}{2} \mu_{2} (\overline{I}_{2} -3), $$

where *μ*=*μ*_1_+*μ*_2_ is the shear modulus of the material. The terms $\overline {I_1} = \text {tr} \, \overline {\mathbf {C}}$ and $\overline {I_{2}} = 1/2 \left [ (\text {tr} \, \overline {\mathbf {C}})^{2} - \text {tr} (\overline {\mathbf {C}}^{2}) \right ]$ are the first and the second invariants, respectively, of the isochoric Cauchy-Green deformation tensor $\overline {\mathbf {C}} = \overline {\mathbf {F}}^{T}\overline {\mathbf {F}}$, with $\overline {\mathbf {F}} = J^{-1/3}\mathbf {F}$. The anisotropic term *Ψ*_aniso_ models two statistically dispersed families of collagen fibrils (about 40% of the total collagen), which confer an orthotropic nature to the material. The distribution of the fibril family *M*, assumed to be of von Mises type, is defined in terms of a unit vector field, **a**_*M*_(**x**), identifying the main orientation of the fibrils, and of a dispersion coefficient *b*_*M*_(**x**), cf. [16]. The anisotropic strain energy function *Ψ*_a_ used in the model is

$$ \begin{aligned} \Psi_{\text{aniso}} ({I^{*}_{4}{M}}, \boldsymbol{\sigma}_{M}) = &\sum_{M=1}^{2} \frac{k_{1\,M}}{2k_{2\,M}} \exp \left[k_{2\,M} \left({I^{*}_{4}{M}} - 1 \right)^{2}\right]\\ &\left(1 + K_{M}^{*}({I^{*}_{4}{M}}) \boldsymbol{\sigma}_{M} \right), \end{aligned} $$

where *k*_1 *M*_ is a stiffness parameter that controls the fibril behavior at moderate extension, and *k*_2 *M*_ is a dimensionless rigidity parameter that regulates the fibril behavior at large extension. The pseudo-invariants $I^{*}_{4}{M}$ are defined as

$$ {\begin{aligned} {I^{*}_{4}{M}} \!&=\! {\mathbf{H}}_{M} : {\mathbf{C}} \,, \qquad {\mathbf{H}}_{M} = \langle {\mathbf{A}}_{M} \otimes {\mathbf{A}}_{M} \rangle = \kappa_{M} {\mathbf{I}} + (1 - 3 \kappa_{M}) \,,\\ \qquad {\mathbf{A}}_{M} &= {\mathbf{a}}_{M} \otimes {\mathbf{a}}_{M} \,. \end{aligned}} $$

The scalar parameter *κ*_*M*_ depends of the spatial distribution density, *ρ*_*M*_(*Θ*), of the fibril orientation. According to the chosen distribution density, the material model can describe full 3D transversally isotropic sets of fibers [35]. The expression of *κ*_*M*_ is

$$ \kappa_{M} = \frac{1}{4}\int_{0}^{\pi}\rho_{M}(\Theta)\sin^{3}\Theta d\Theta \,. $$

The two terms

$$ \begin{aligned} K_{M}^{*}({I^{*}_{4}{M}}) &= k_{2\,M} + 2 \,k_{2\,M}^{2} \, \left({I^{*}_{4}{M}} - 1\right)^{2},\\ \qquad \boldsymbol{\sigma}_{M} &= {\mathbf{C}} : \langle {\mathbf{A}}_{M} \otimes {\mathbf{A}}_{M} \rangle : {\mathbf{C}} - \big({\mathbf{H}}_{M} :{\mathbf{C}} \big)^{2} \,, \end{aligned} $$

account for the variance of the fibril orientation distribution, cf. [35]. The second order approximation of the strain energy function leads to the introduction of additional integral coefficients, which read

$$ \widehat{\kappa}_{M} = \frac{1}{16}\int_{0}^{\pi}\rho_{M}(\Theta)\sin^{5}\Theta d\Theta \,. $$

For details about the derivation we refer to the original works [35, 38, 45] where the corresponding tangent stiffness is also provided.

## Appendix B

The hypothetical distribution of the axisymmetric air jet footprint used in [12, 27, 30] is

9 $$ {\begin{aligned} p_{\text{jet}}({\mathbf{x}}, t) = p_{\text{peak}} \exp(- d \, r^{2}) \exp \left[-b \left(\frac{t}{T} - \frac{1}{2}\right)^{2} \right] \,, \quad {r^{2} < R^{2}} \,, \end{aligned}}  $$

where *r* denotes the current in-plane distance between a point on the anterior surface of the cornea and the center of the air jet, *R* the assigned radius of the air jet circular footprint on the anterior surface, *p*_peak_ the maximum pressure of the air jet, *T* the total duration of the jet, and *b*, *d* parameters that govern the time and in-plane distribution of the pressure.

## Data Availability

All data used in this study, in form of list of nodal coordinates and element connectivity tables, will be made available upon request.
